# Phenotyping for the dynamics of field wheat root system architecture

**DOI:** 10.1038/srep37649

**Published:** 2017-01-12

**Authors:** Xinxin Chen, Qishuo Ding, Zbigniew Błaszkiewicz, Jiuai Sun, Qian Sun, Ruiyin He, Yinian Li

**Affiliations:** 1 Key Laboratory of Intelligent Agricultural Equipment of Jiangsu Province, College of Engineering, Nanjing Agricultural University, Nanjing 210031, China; 2 Institute of Biosystems Engineering, Poznań University of Life Sciences, Poland; 3 Shanghai University of Medicine & Health Sciences, 101 Yingkou Road, Yangpu district, Shanghai, 200093, China

## Abstract

We investigated a method to quantify field-state wheat RSA in a phenotyping way, depicting the 3D topology of wheat RSA in 14d periods. The phenotyping procedure, proposed for understanding the spatio-temporal variations of root-soil interaction and the RSA dynamics in the field, is realized with a set of indices of mm scale precision, illustrating the gradients of both wheat root angle and elongation rate along soil depth, as well as the foraging potential along the side directions. The 70d was identified as the shifting point distinguishing the linear root length elongation from power-law development. Root vertical angle in the 40 mm surface soil layer was the largest, but steadily decreased along the soil depth. After 98d, larger root vertical angle appeared in the deep soil layers. PAC revealed a stable root foraging potential in the 0–70d period, which increased rapidly afterwards (70–112d). Root foraging potential, explained by MaxW/MaxD ratio, revealed an enhanced gravitropism in 14d period. No-till post-paddy wheat RLD decreased exponentially in both depth and circular directions, with 90% roots concentrated within the top 20 cm soil layer. RER along soil depth was either positive or negative, depending on specific soil layers and the sampling time.

Crop root systems still remain as an underexplored target for improvements of crop yields and productivity^1^. A promising finding for crop root systems is that increased yield and productivity is attainable with improved root system architecture (RSA) traits, being explained as an optimized spatial configuration of root structures in the soil^2^,^3^. Although the importance of crop RSA in determining the uptake and transport of water and nutrients has been addressed in a number of experiments^3^,^4^ and simulated with numerous mathematical models^5^,^6^, our knowledge on the dynamical process of crop RSA development, particularly in its field state, still remains quite limited.

The crop RSA dynamics in field is difficult to access, identify and quantify. The challenge for root studies is to improve techniques and methods for root sampling and root system architecture quantification^7^. A rich number of sampling and phenotyping methods for crop roots in the field have been proposed, including soil profiles^8^, monoliths^9^, nail plates, probes^10^, rhizotron^8^, trenching^11^, shovelomics^12^ and digitalization and visualization of roots in field^13^,^14^. These methods supply a number of parameters, e.g. root dry matter, root length and diameter, root surface area, root dry weight, root diameter classes and root structure^15^,^16^. Despite these tremendous efforts, the shape of entire root system is still unclear to us ref. 3. Extrapolation of these phenotyping tools to the field-state crop RSAs is even scarcer. Field crop RSA phenotyping is hampered, not only by the biological, chemical, and physical complexities of the soil medium^17^ but also by a shortage of accurate and comprehensive information about root systems and how they work throughout the lifespan of plants in the field^18^. These are also the most critical aspects for modeling roots and for identifying root architectures suitable to agricultural or natural systems^19^.

Despite its richness, the capacity for undertaking precision phenotyping, particularly under repeatable and representative growing conditions in the field, is lagging far behind genomics^20^,^21^. Quantifying crop RSA is important because crop productivity is almost always influenced by the interplay between RSA and soil nutrient availability^22^. A better understanding of root phenomena is also critical for crop cultivars^23^, the key of which should be based on detailed description of field-state crop RSAs. Spatially distributed monolith sampling is an option for illustrating crop RSAs with root length and root mass spatial heterogeneities in all three spatial dimensions in field^9^. Whereas commonly applied auger core sampling can incur large errors when characterizing spatial distributions of roots^24^. Shovelomics, another high-throughput method for phenotyping RSAs in the field, provides a quick sampling and quantification solution^12^. But its simplicity is achieved by sacrificing the detail of RSAs.

Optimization of a protocol for field crop RSA quantification requires proper design on both hardware and software for root sampling, digitizing, visualizing and analyzing. Increased numbers of high-throughput phenotyping platforms have been developed in recent years, most of which were run by the big transnational seed companies and the most advanced public plant research institutions around the world^20^. However, low-cost phenotyping approaches are now starting to be developed^25^. Instead to the above-ground phenotyping, Chen *et al*.^14^ proposed a method particularly suitable for crop RSA in the field, especially for fine and fibrous root systems. The approach differed largely from modeled crop RSAs in that all the RSA analyzing and presentation is a data-driven procedure with no simplification on the boundary conditions of the root-soil interactions. It thus guarantees that the analyzed RSAs conform to the real-world root system topology in the field. This paper aims to investigate more advanced calculations of crop RSA parameters, through which the behavioral dynamics of field crop RSAs could be highlighted.

## Results

### Distribution of wheat RSA

#### Root length dynamics

**Figure 1:**
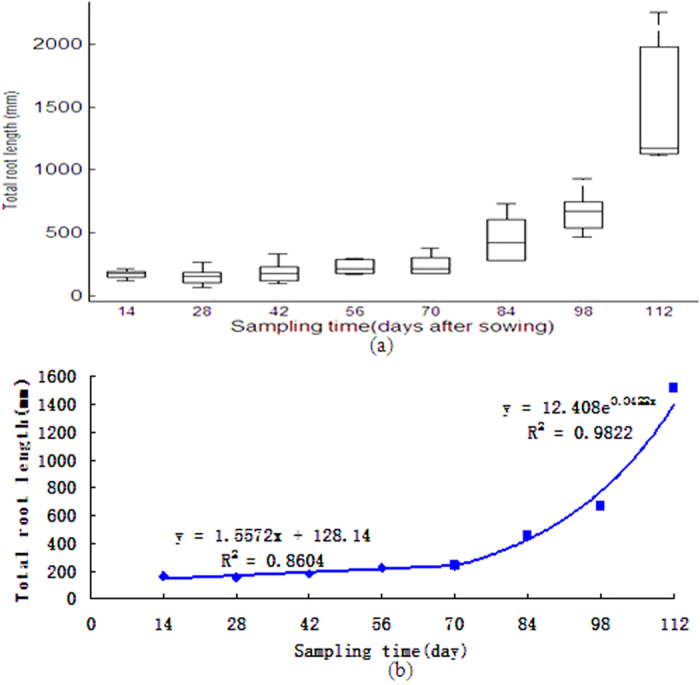
Dynamics of root total length of wheat RSAs. (**a**) box plot, (**b**) curve fitting of the 2 root elongation periods.

Figure 1a illustrates the total root length variations in each period. A steady linear increasing stage of root length was observed during the first 70d, which was ensued with a rapid surge of root elongation. Hund *et al*.^26^ found that the length of axial roots of the seedling stage had a linear increasing trend. Barraclough *et al*.^27^ investigated the later stage of root development and found a power law function applicable to a stage until the anthesis period. Our findings in the post-paddy wheat field confirmed the validity of both these trends. However, it would be more meaningful to identify the instance on which the transition from linear elongation to the power law function was initiated. Identification of this shifting point as a key physiological time-node is important for precision field crop management, e.g. topdressing or irrigation. Root length dynamics in Fig. 1b clearly shows the 70th day is an accurate time-node on which it should be concerned. More detailed information is presented in supplementary text S2 and D1.

Wheat root systems do not elongate in a same rate throughout its lifecycle, a feature of importance when evaluating root functions and the influence of soil on them^28^. Understanding both the process of growth and the life-cycle dynamics of root systems is essential when precision management of root system and soil function is necessary^29^. As topologically identical root systems can still take on very different appearances if they vary in metric aspects of their geometry^2^, topological interpretation on the dynamics of RSA (e.g. root angle and root elongation rate) is more critical.

#### Root vertical angle dynamics

**Figure 2:**
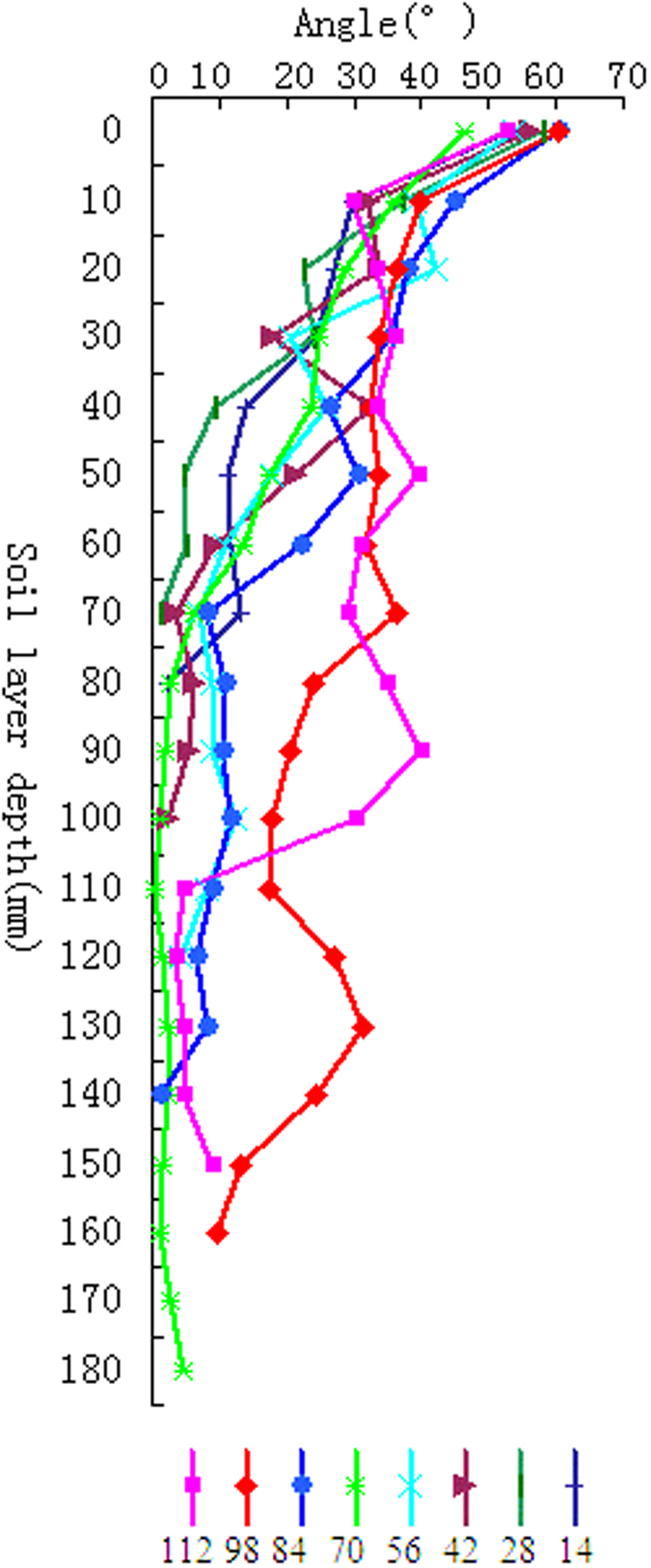
Dynamics of root vertical angle. (Numbers of the legend (14, 28, 42, 56, 70, 84, 98 and 112) stand for DAS).

Figure 2 shows that not only root vertical angles changed along the depth, but also they varied in each sampling period. More detailed information is presented in supplementary text S2 and D2. In all stages root vertical angle was the largest in the surface soil layer (0–40 mm) and decreased rapidly along soil depth. Similar result was also found by Lynch and Brown^22^, who indicated that roots with large vertical angles would predominantly explore the topsoil for nutrition. In the latter stages (e.g. 98 d and 112 d) this trend was invalidated, a feature has never been reported before. This wheat RSA dynamics clearly illustrates that the mechanisms of root-soil interaction in the field is unique, which could not be found in the lab cultivated crop RSAs.

Root angle was identified as an important determinant for spatial colonization of roots^30^. The trajectory of an axile root in the soil is initially determined by the root angle, which is primarily governed by plagiogravitropism^31^. Root vertical angle and its dynamics explain the mechanisms governing the root-soil interactions, e.g. roots with small vertical angles will predominantly explore deeper soil layers for water^32^. Nieuwenhuis *et al*.^33^ indicated that changes of soil depth, variation of soil penetration resistance or a presence of a plow pan can significantly affect root angles. Additional factors, such as soil temperature^34^, soil water status^35^ and levels of phosphorus^36^, nitrogen^37^ and soil strength^31^ also play important roles in affecting root angle Identifying individual factor from the coupling effects is not possible for the time being, particularly for field-state crops. However, Fig. 2 at least provides us an initiation to illustrate the detailed root angle dynamics along soil depth, depicting the interplay between the root and the soil environment. Phenotyping for 3D field-state crop RSA is clearly distinctive from traditional washed root methods either carried out manually^38^ or analyzed with image processing tools^39^. A basic fact of those traditional methods is that original 3D crop RSA were totally destroyed, with no remnant signature of RSAs^40^.

#### Percentage area coverage dynamics

**Figure 3:**
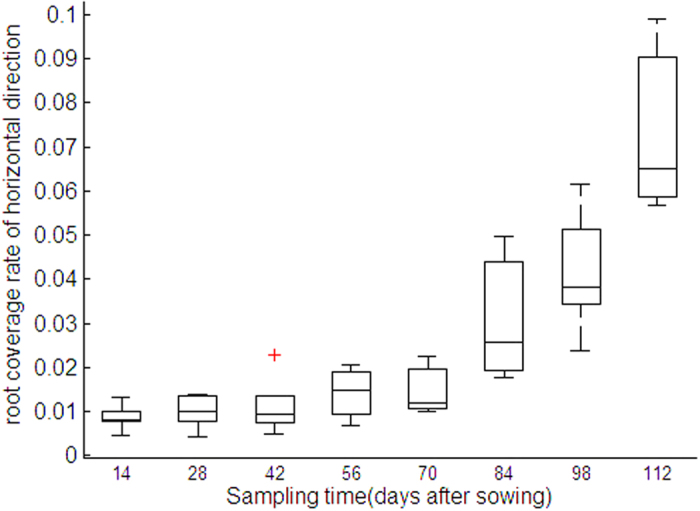
Percentage horizontal soil coverage by root.

Percentage area coverage (PAC) parameterizes root horizontal distribution in the growing media. Figure 3 illustrates the dynamics of wheat root PAC in each period, carrying the similar trend of dynamics with the total root length. Root distribution remains stable during the winter period (0–70 d) and increases rapidly in later stages (70–112 d). More detailed information is presented in supplementary text S2 and D3.

Modeled wheat RSAs were virtually reconstructed in Pro-E with spline curves, carrying no information of foraged soil volume or area coverage by the crop RSAs. Root foraging potential can be parameterized with meshing analysis on the projected RSA in the 2D viewport, in which the RSA projection was meshed in a 2.5 mm resolution. This meshing tactic was referenced to the well established conclusion that effective zone of root uptake of immobile nutrient was restricted to 2–4 mm^41^. Ahmed *et al*.^42^ found that active zone of root P absorbing was only in several mm range. A distinctive feature of the calculated PAC with Pro-E is that it illustrates the horizontal root distribution of an individual plant instead of the whole a crop community obtained by core sampling or trenching. Root horizontal distribution can also be quantified with MaxW of RSAs. Clark *et al*.^30^ measured MaxW in every 0.2° rotation in the lab. However, crop RSA cultivated from greenhouse can have significant difference from those field-grown.

#### MaxW/MaxD ratio

The dynamics of MaxW/MaxD ratio is helpful to illustrate how environmental factors (e.g., water distribution in the soil layers, temperature gradient or soil hardness variations) may contribute to the principal mechanisms affecting crop RSA processes.

**Figure 4:**
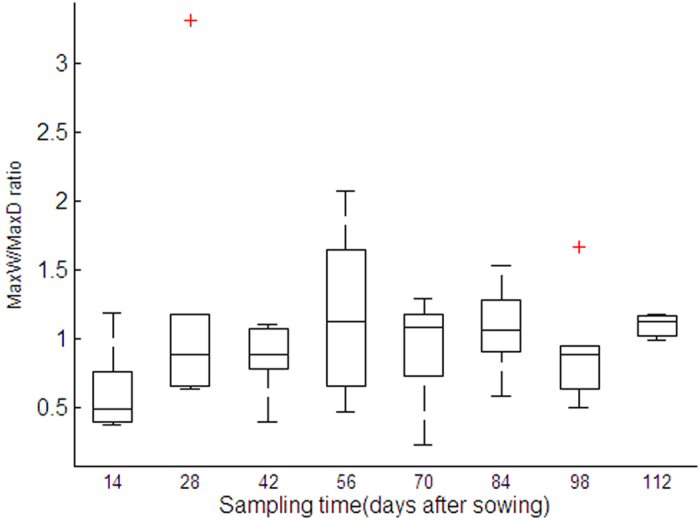
Dynamics of MaxW/MaxD ratio (ratio of maximum width to maximum depth of wheat RSA). MaxW/MaxD ratio describes whether root system is preferentially elongated horizontally or vertically, a unique feature of root-soil interactions. MaxW/MaxD ratio explains whether the root system development is shallow or deep, and whether gravitropism or thigmotropism plays the decisive role.

Figure 4 reveals that root elongation experienced an alternated mode of shift between gravitropism and laterality (i.e., the tendency of root horizontal elongation). The 14 d period was featured an enhanced gravitropism, followed with a period of lateral root elongation. This alternation dampened progressively in later stages. More detailed information is presented in supplementary text S2 and D4. Related research found that root foraging in surface soil layer increases the water and nutrient utilizing efficiency^22^. However, by which mechanism this RSA alternation is governed, e.g. through hydraulic, nutrient or soil physical factors etc., is not known. When combining with Figs 1 and 3, it may be induced that increased root length or foraged soil volume provides an enhanced damping effect on the dynamics of MaxW/MaxD ratio.

Crop RSA dynamics is attributed to three intrinsic growth responses: circumnutation, gravitropism and negative thigmotropism^43^. Plants in the field experience a range of stresses throughout their life cycle. In many cases, the environmental characteristics are not monitored and, hence, are poorly understood^20^,^21^. Phenotyping for the dynamics of RSA can be a useful tool to interpret the interplay between the root and field conditions. Environmental variability inconsistently affects phenotypic observation over both space and time and must be accounted for in any statistical models that are used to estimate parameters of interest^5^. The fluctuation of the observed MaxW/MaxD ratio means that periodic infiltration or water distribution dynamics among soil layers modifies the depth or sideway elongation potential of root system, which mechanism still waits for further investigation.

### Soil colonization by wheat RSA

#### The gradient dynamics of RSA

Greater root length density (RLD) is an effective strategy adopted by the plant for improved nutrient acquisition by increasing root surface areas^39^. RLD gradient along a particular direction explains the potential of the root exploiting in that orientation, and thus is an indicator of root foraging priorities either governed by crop physiologies (e.g., gravitropism) or modified by the soil environment (e.g., nutrient distribution or water availability). The gradient of RLD is analyzed in both horizontal and vertical directions, indicating how effective the root system could extend in a particular direction into the surrounding soil^33^.

**Figure 5:**
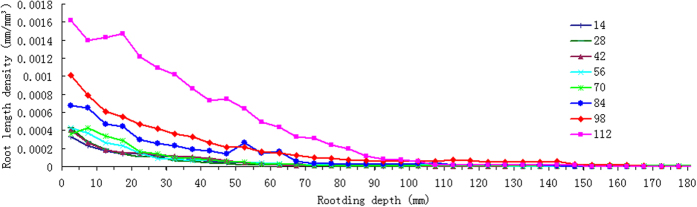
Root density gradients along soil depth.

**Figure 6:**
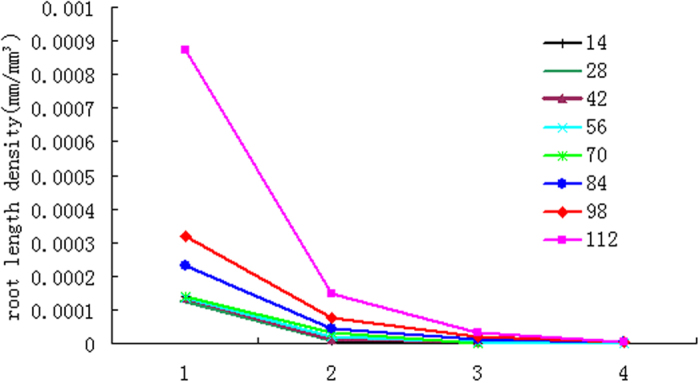
Circular root density gradients. 1 Represents section one roots; 2 represents section two roots; 3 represents section three roots; 4 represents section four roots.

The post-paddy wheat RLD was found decreasing exponentially in both depth and circular directions (Figs 5 and 6). More detailed information is presented in supplementary text D5 and D6. Unlike most of the proposed single decaying functions reported in related studies, as those summarized by Mao *et al*.^44^, our results revealed that RLD gradient of the post-paddy wheat also changes in different stages of crop development: the later the crop stages, the larger RLD decaying potential.

Constraints on no-till post-paddy wheat RSA were obvious, with around 90% roots concentrated within the top 20 cm soil layer (Fig. 5). The sampling depth for RDW also reported that 80–90% of the total RDW is distributed in the top 0 to 20 cm soil layer^45^. Root development in the horizontal direction was confined within a radius of 60 mm (Fig. 6), with a majority of it confined within a cylinder in 40 mm diameter soil cylinder. In the first 70d period, wheat root was restricted in a shallow soil layer. In later stages (70 d–112 d), particularly in tillering and jointing periods, the root was found penetrated into deeper soil layers, but only a limited number of axial roots. This obvious restriction explained by RLD gradients indicates that the wheat grown in the paddy soil is largely affected by the adverse soil physics. Related studies have indicated that, for wheat in the rice-based cropping rotations, soil physical conditions created by wet tillage for rice (i.e., puddling) are widely considered as a key reason for the gap between potential and realized levels of productivity^46^,^47^. Introduction of no-till to the rice-wheat rotation generally results into increased yields of wheat as compared to conventional tillage under constrained resources^48^. The restricted RSA is clearly an underlining factor governing the mechanisms for degraded wheat performance in the rice-based crop rotations in China.

Increased soil volume exploration, as a result of continuous root branching, goes hand in hand with enhanced RLD^49^. A root system distributed non-uniformly can extract water and nutrients much differently. A system with larger RLD absorbs water and nutrients faster^50^. RLD gradient also exists across the row in maize crops^51^, the variation of which with distance from the plant was qualitatively similar both to our findings and to that found by Gajri *et al*.^51^. The RLD was much greater near the plant base in the surface soil layer. In deeper layers, this variation becomes smaller. Wheat RLD gradient was also affected by soil texture^51^.

Deeper root system is associated with early vigor of wheat, which is often a soil dependent trait and could be best utilized under specific growing conditions^52^. The apparent constraint on post-paddy wheat RSA means that available nutrients may concentrate in a shallow soil layer when the paddy soil is no-tilled. Some other researchers have emphasized the importance of quantifying circular expansion of crop root, as it not only reflects the adaptive behavior of crop root to the environment, but also stands for the ability of root in exploiting the soil volume^53^. Quantifying the circular expansion of crop root provides a basis for reasonable seeding density^54^,^55^. The large gradient of RLD in its circular direction means that narrow row spacing is necessary for no-till post-paddy wheat cultivation.

#### Wheat root elongation rate along the depth

**Figure 7:**
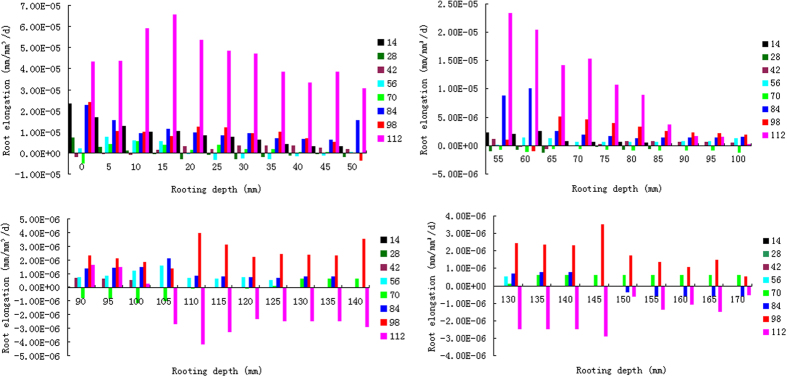
Dynamics of wheat root elongation rate along soil depth.

Root elongation rate (RER) is a pivotal parameter of crop RSA, as it explains the difference of root length between adjacent sampling periods. RER explains whether the RSA is flourishing or perishing in a particular soil layer. Improved growth rates of axial roots to the depth may be exploited for a better acquisition of water from deep soil layers^56^ or a better N efficiency^57^. In our experiment, RER along soil depth was either positive or negative, depending on specific soil layers and the sampling time (Fig. 7). Positive RER was occasionally observed in shallower layers. In deeper soil layers, however, withered root system was found in some periods, including the 70, 84 and 112 DAS. The identified 3 periods of root withering revealed a shifted depth from time to time. Root perished in 90–110 mm soil layer in the 70 DAS, but in 150–180 mm in the 84 DAS, while in the 112 DAS, it happened in 105–170 mm layer. However, instead of the root perishing in deeper soil layers, the 112 DAS showed an intensive root flourishing in the shallow soil layers (0–100 mm), revealing an intense foraging activity in this period. Positive or negative RER also means that there are complicated hidden mechanisms that govern the foraging behavior of wheat root system waiting for future investigation. More detailed information is presented in supplementary text D7.

## Discussion

Phenotyping is not only a key for genomics, but also serves as an important tool for explaining the mechanisms and processes of the interplay between crop root system architecture and the soil environment. Field conditions are notoriously heterogeneous and the inability to control environmental factors makes the collected results difficult to be interpreted. Current results from controlled environments are far removed from the situation plants will experience in the field and, therefore, are difficult to extrapolate to the field^20^,^21^.

The analyzed wheat RSAs with Pro-E were mirrored pairs with its *in-situ* field-state topologies, without any imposed modification. This fact guarantees that this phenotyping tool identifies all the field-level phonologies and fingerprints of the soil-root interactions^14^. Parameterization of crop RSA and illustrating with the time-series presentations provides a useful means of phenotyping for the geometric, topological and distribution behaviors of wheat RSAs in the field.

Crop root distribution can be a response to phosphate availability, which reflects a rooting strategy for fixed nutrient foraging^22^,^36^. Effective root uptake of phosphate is confined within mm scale^41^,^42^. In Pro-E modeling, soil volumetric effectiveness of wheat root was analyzed in respective mm scales. Both root length and root horizontal distribution increased progressively (Figs 1 and 3), indicating an enhanced nutrient uptake along the time. Changes of mean root angle across soil depth illustrate the topological features of RSAs (Fig. 2). This presentation avoids root washing by the traditional methods of root measurement, which led to profound modification on the original state of RSA and made it un-applicable for fine and fibrous root system quantification^40^. The MaxW/MaxD ratio (Fig. 4) reflects whether a plant develops shallow or deep root systems. In this study, the post-paddy wheat was found to have a shallow root system, which may be accounted by the physical restriction from the soil. Root vertical angle during 0–84 d was relatively stable, which was consistent with previous findings^12^,^58^. Root angle could be affected by soil heterogeneities^31^. Soil environment variation has a weighted influence over the dynamic readjustments of root allocation, morphology, and spatial deployment^59^. This could be an account for the observed changes of root angle during 98–112 d. Lynch and Brown^22^ indicated that high root distribution in the upper soil layer would be advantageous for efficient use of surface-applied nutrients. Miao *et al*.^60^ found that depth distribution of crop root is not genetically governed, but modified by the growing media and controlled by the environmental factors. The saturated soil state during the paddy season provides a more fragile environment for the soil structures, which is more sensitive to compaction and condensation or hard-setting. Annual rice-wheat rotation and repeated traffic wheeling resulted into a dense plow pan in the paddy soil^61^. If the soil is compacted to certain degree it may prevent roots growing through it and force the roots to exploit through cracks and pores, reducing the random nature of the root distribution^62^. Unfortunately, most of these findings were descriptive results. The phenotyping method quantified the extent of paddy soil restriction on wheat root elongation, revealing the contrasted finding from dryland wheat, e.g. Wang *et al*.^63^, who found that the root systems extended to 85 ± 16 cm from the soil surface.

Soil monolith^64^ is a commonly used method for the special distribution of crop roots, which samples the soil in a three-dimensional spatially distribution monolith scheme^8^. The monoliths sliced the soil volume into 5 or 10 cm increments along the depth and the root length in each soil block were measured^9^,^64^. No report has appeared for monolithing the crop RSA in a finer resolution of mm scales. The phenotyping method proposed in this paper was applied to individual plant and analyzed crop RSA in mm scale (Figs 5, 6 and 7), vividly illustrating how wheat root behaved across each soil layers. For a non-uniformly distributed root, an area where the root density is larger also means a much faster water and nutrients uptake^50^. Resources could be largely inaccessible in regions of the soil where rooted sparsely. The superposition of soil and root data in 3D space promises to give new insight into how roots explore the soil environment. As the increase of root density is a potential contribution to crop yield^39^, the phenotyping protocol for wheat RSA provides a suitable means to illustrate the interplay among root, soil and the environment, and serving the precision agriculture for a better crop management.

## Methods

### Site description

Winter wheat (Ningmai13) was grown in a paddy field after rice harvesting in Jiangpu Experimental Farm, Nanjing Agricultural University, China. The site is located at 31°98′N, 118°59′E, in subtropical monsoon climate, with an annual rainfall of 1048.6 mm and a mean temperature of 15.8 °C^65^. Annual rice-wheat rotation is the traditional cropping system in the local region. The paddy season begins in June and ends up by the end of November. A month before rice harvesting the field is drained, allowing the soil to turn into dry state for mechanical harvesting^66^. On 20 November 2011 wheat was sown into non-tilled soil in small plots (5 × 3 m^2^) in 3 replications. Wheat seeds were manually placed uniformly on the soil surface in a 5 × 5 cm^2^ grid pattern to mimic no-till surface broadcasting, a practice widely adopted in Sichuan Province, China^67^. More detailed information is presented in supplementary text S1. The uniform placement of seeds also guarantees a minimised plant-to-plant interaction^68^,^69^, which has potential effects on RSAs. Seeded plots were covered with a thin layer of fine soil.

Soil organic matter, total N, available N, available P and available K in the field were tested to be 8.24 g∙kg^−1^, 0.97 g·kg^−1^, 12 mg∙kg^−1^, 12.67 mg·kg^−1^ and 11.05 mg∙kg^−1^, respectively. Soil pH, bulk density and water content were tested to be 7.6 and 1.26 g∙cm^−3^ and 29.3%, respectively. Phosphate, urea and potassium chloride were applied to the soil surface at amounts of 375 kg∙hm^−2^, 90 kg∙hm^−2^ and 375 kg∙hm^−2^, respectively. The whole wheat is rain-fed and the crop is managed in the same way as the local farmers do.

### Wheat root sampling and measurement and modeling of RSA

Root zone soil was sampled on 14, 28, 42, 56, 70, 84, 98 and 112 days after sowing (DAS). Two plant roots per plot were excavated and totally six replicates were collected in each sampling period^12^,^70^. In the last time only 3 wheat roots were collected, due mainly to the overcrowding of the roots and the overwhelming time required for digitizing. Only those plants with average appearance in the field were sampled and analyzed.

A large soil core in 16 cm diameter and 25 cm height was positioned concentric to the base of the plant stem, and driven into the soil with a hand hammer. The core with the soil and the undisturbed wheat root system was excavated with a shovel and was brought to the laboratory for digitizing. Digitizing of wheat RSAs was performed with an adapted digitizer and in a layered excavating procedure as described by Chen *et al*.^14^ and then used for wheat RSA modeling, from which undisturbed wheat RSAs were digitized and the collected data were transferred to Pro-E for modeling.

### Parameterization of modeled RSAs

**Figure 8:**
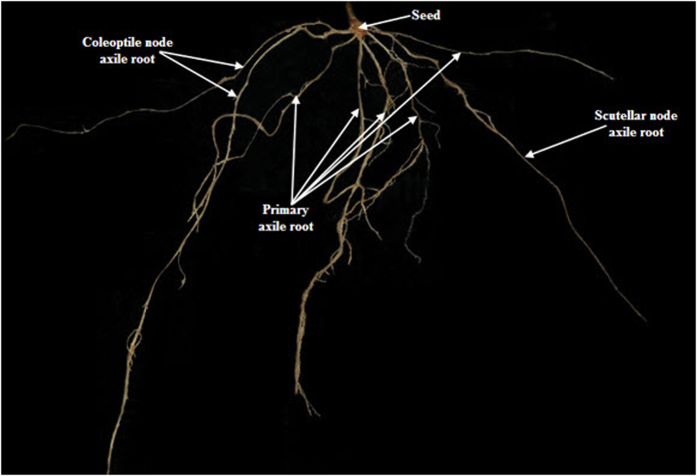
Root system of Ningmai 13 with primary and nodal axile roots.

Topological structure of a wheat root system is composed of both seminal roots (those that develop early and originate from structures in or very close to the seed) and adventitious, nodal or crown roots (those that originate later from nodes of the stem)^71^ (Fig. 8). Parameterization of crop RSAs needs a set of indices, including root length, root angle, maximum width of root distribution (MaxW), maximum depth of root distribution (MaxD), MaxW/MaxD ratio, horizontal soil coverage ratio, root vertical distribution, root/soil ratio in horizontal and vertical directions, and so on.

#### Total root length

Total root length of each plant is calculated with an embedded computing module in Pro/E^14^. The mean of the samples quantifies the RSA development status.

#### Maximum RSA width (MaxW)

**Figure 9:**
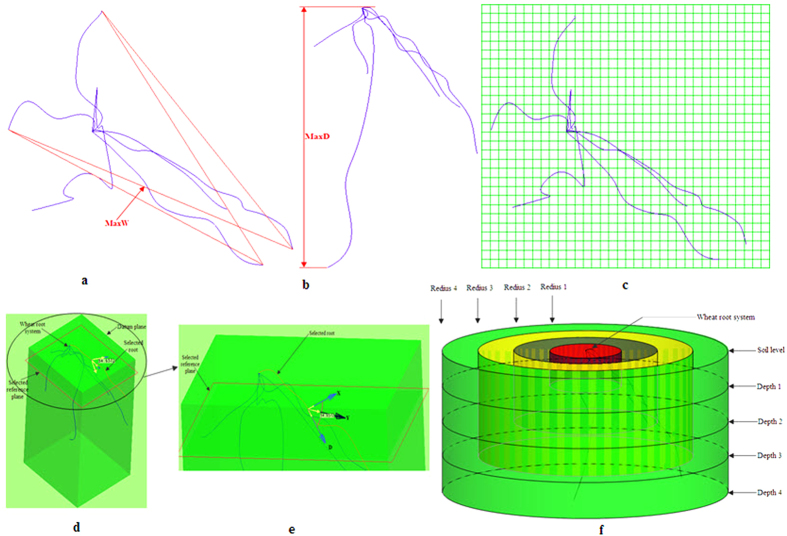
Measurement of root in Pro-E. (**a**) Represents the measurement of MaxW, (**b**) represents the measurement of MaxD, (**c**) represents the measurement of percentage area coverage, (**d**) represents a root overall structure and (**e**) represent a local magnification of (**d** and **f**) represents the measurement of root density gradient).

MaxW is the maximum horizontal width of the whole root system or root system component. MaxW was measurable only on the imaged RSAs, e.g. a statistical count on the maximum width by rotating every 0.2° of the horizontal image of a root system^30^. In this research, the 3D RSA model was vertically projected to a level reference plane in Pro-E, then sketch in this plane and draw lines (Fig. 9a). In the last step, the distances between every two root tips were measured through embedded analytical tools of Pro-E, i.e. Analysis-Measure-Length, the maximum of which is MaxW.

#### Maximum RSA depth (MaxD)

MaxD of a root system was measured in relation to the upper-most slice containing a root system voxel (Fig. 9b)^30^.

#### MaxW/MaxD ratio

MaxW/MaxD is defined as the ratio of maximum width to maximum depth.

#### Percentage area coverage (PAC)

The modeled wheat RSA was projected to a level reference plane, which was 160 mm in size and was split into 64 × 64 grids (Fig. 9c). The numbers of cells containing root sections were counted and the ratio of the counted cells to the whole is defined as horizontal PAC:


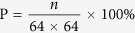


where *n* is the number of cells containing root sections, *p* is horizontal PAC by root.

#### Root vertical angle

Root vertical angle is the direction of a root section with respective to the horizontal plane^72^. Pro-E provides a means to determine this angle by measuring the orientation of a root section with reference to horizontal plane (Fig. 9d). The horizontal plane containing the datum point (also the seed point) was designated as a datum plane (soil level), from which a series of parallel reference planes were produced along Z axis with a 10 mm distance from each other. These reference planes intercept with the virtual RSA, resulting into a series of root sections within each layer. Root vertical angle was calculated as the intersection angle between the tangent line D of the root section and the reference plane in each layer (Fig. 9e). This calculation was also performed automatically in Pro-E with limited commands executing the following steps: Aanlysis-Measure-Angle followed by choosing the target root and reference plane and execute. The mean of all the calculated angles within a layer is the’mean root vertical angle’for this layer.

#### Root density gradient

Root density gradient (RDG) is the change of RLD in a particular orientation. The depth RDG is quantified as the change of RLD (or root/soil ratio) in the depth direction. Similarly, the sideway RDG is the change of RLD along the peripherals. The depth RDG measurements were taken based on imaginary cylinders (Fig. 9f) and the modeled wheat RSA was sliced into successive layers, with 5 mm distance between adjacent layers. The total length of the root sections in each layer was calculated with embedded functions in Pro-E. Wheat RSA was managed in the Front datum plane, which was divided into a series of sections, each is 5 mm thick. Sequentially execute Edit and Trim command to section the RSA into separate layers. Now following Analsis-Measure-Length commands and the total length of root sections within this layer is calculated. The mean of the total length of all the root sections within a layer was taken as the mean total length of the layer i.e. L_i_ (i = 1, 2, 3…). Soil volume of each layer is calculated as the cylinder volume a 160 mm diameter and a 5 mm height. RLD of the i^th^ layer is calculated as L_i_/V. Relative root elongation rate (RER) (mm^−1^ day^−1^) was calculated as the subtraction of the mean total root length in each depth with that from the last time and averaged by 14 days. Relative root elongation rate explains whether the root system is flourishing or perishing in a particular soil layer and during a particular period.


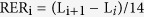


where RER_i_ is the relative root elongation rate of the i^th^ layer, L_i+1_ is current mean root length of the i^th^ layer, L_i_ is the mean root length of the i^th^ layer in the last time.

The circumferential RDG is calculated as the change of RLD along the sideway orientations, and the measurements were based on four imaginary cylinders (Fig. 9f). The radii of the four cylinders were 20, 40, 60 and 80 mm, respectively. All roots located inside the first cylinder were termed section one roots. Roots falling inside the second cylinder, but outside the first cylinder were called section two roots, and so forth. Root length of each section was calculated and the mean of all the samples in each section was determined as the circumferential RDG.

## Additional Information

**How to cite this article**: Chen, X. *et al*. Phenotyping for the dynamics of field wheat root system architecture. *Sci. Rep.* **7**, 37649; doi: 10.1038/srep37649 (2017).

**Publisher's note:** Springer Nature remains neutral with regard to jurisdictional claims in published maps and institutional affiliations.

## Supplementary Material

Supplementary Information

Supplementary Data
