# Supplementary material for: Phenotyping for the dynamics of field wheat root system architecture
Source: Sci Rep. 2017 Jan 12;7:37649. doi: 10.1038/srep37649 (PMC5227993; doi:10.1038/srep37649)
Supplement: Supplementary Information [file srep37649-s1.pdf]

Phenotyping for the dynamics of field wheat root system architecture

Xinxin Chen[1], Qishuo Ding[1*], Zbigniew Błaszkiewicz[2], Jiuai Sun[3], Qian Sun[1], Ruiyin He[1], Yinian Li[1]

1 Key Laboratory of Intelligent Agricultural Equipment of Jiangsu Province, College of Engineering, Nanjing Agricultural University, Nanjing 210031, China

2 Institute of Biosystems Engineering, Poznań University of Life Sciences, Poland

3 Shanghai University of Medicine & Health Sciences, 101 Yingkou Road, Yangpu district, Shanghai, 200093, China

First author: Xinxin Chen, E-mail address: lingyinyu@163.com

Corresponding author: Qishuo Ding, E-mail address: qsding@njau.edu.cn, telephone number: 15850723939, fax number: 0086-25-58495897

Third author: Zbigniew Błaszkiewicz, E-mail address: zbigniewblaszkiewicz@up.poznan.pl

Fourth author: Jiuai Sun, E-mail address: sunja@sumhs.edu.cn

Fifth author: Qian Sun, E-mail address: wendysunnjau@sina.com

Sixth author: Ruiyin He, E-mail address: ryhe@njau.edu.cn

Seventh author: Yinian Li, E-mail address: liyinian@163.com

Abstract

We investigated a method to quantify field-state wheat RSA in a phenotyping way, depicting the 3D topology of wheat RSA in 14d periods. The phenotyping procedure, proposed for understanding the spatio-temporal variations of root-soil interaction and the RSA dynamics in the field, is realized with a set of indices of mm scale precision, illustrating the gradients of both wheat root angle and elongation rate along soil depth, as well as the foraging potential along the side directions.

The 70d was identified as the shifting point distinguishing the linear root length elongation from power-law development. Root vertical angle in the 40 mm surface soil layer was the largest, but steadily decreased along the soil depth. After 98d, larger root vertical angle appeared in the deep soil layers. PAC revealed a stable root foraging potential in the 0-70d period, which increased rapidly afterwards (70-112d). Root foraging potential, explained by MaxW/MaxD ratio, revealed an enhanced gravitropism in 14d period. No-till post-paddy wheat RLD decreased exponentially in both depth and circular directions, with 90% roots concentrated within the top 20 cm soil layer. RER along soil depth was either positive or negative, depending on specific soil layers and the sampling time.

Keywords

Phenotyping, post-paddy wheat, root system architecture, spatio-temporal variation

Financial support:

“ The State Key Special Program of Soil Fertility Improvement and Cropping Innovation for High Yie ld with High Efficiency in Rice Cropping Areas” (Number: 2016YFD0300900).

“Agricultural Machinery Fund of Jiangsu Province” (201-051028)

Author Contributions Statement

Xinxin Chen, Qishuo Ding wrote the main manuscript text. Zbigniew Błaszkiewicz and Jiuai Sun improved the text and provided suggestions on the structure of the manuscript. Qian Sun, Ruiyin He and Yinian Li provided support to the experimental work in the field.

Competing Financial Interests statement

The corresponding author declares that there is no competing interests exist in relation to the work described on behalf of all authors of the paper.

S1

Above ground phenologies of different density

Seed-to-seed distance (CM)	Germination rate (num. m-2)	Mean tiller num.	Ear num.Mu-1	Yield (kg.Mu-1)
M4	439.00a	2.33	3.11E+05	263.03
M5	311.00b	3.05	2.38E+05	301.44a
M6	232.00c	3.91	1.82E+05	274.18c
M7	163.00	4.53c	1.73E+05	234.05
M9	106.00	5.60b	1.36E+05	246.40

*Mu is the Chinese unit of arable land size. The distance of 4 cm, 5 cm, 6cm, 7 cm and 9 cm distances corresponding to 625, 400, 289, 196 and 121 seeds/m^2 respectively.

S2

Statistical analysis on each parameter

Sampling time (day)		14	28	42	56	70	84	98	112
Total root length	variance	34.680	68.699	86.578	52.623	82.347	191.197	166.503	641.404
	mean value	166.353	152.934	184.913	223.373	240.139	453.195	666.667	1510.273
Root vertical angle	variance	4.742	10.390	4.510	8.969	6.538	7.084	6.269	3.064
	mean value	29.281	39.367	36.118	36.896	29.684	34.378	35.137	33.164
Percentage area coverage	variance	0.003	0.004	0.006	0.005	0.005	0.014	0.013	0.022
	mean value	0.009	0.010	0.011	0.014	0.014	0.030	0.041	0.074
MaxW/MaxD ratio	variance	0.311	1.026	0.261	0.615	0.390	0.332	0.405	0.099
	mean value	0.617	1.258	0.858	1.186	0.934	1.073	0.923	1.096

* It seemed that the re-iteration of the plot-shown information with this table won't do much help. The revised plots were more visual and straightforward. Thus I didn't append this statistical table in the manuscript. Provided there is also the conciseness consideration on the manuscript.
